# Prospective multiparametric CMR characterization and MicroRNA profiling of anthracycline cardiotoxicity: A pilot translational study

**DOI:** 10.1016/j.ijcha.2022.101134

**Published:** 2022-11-08

**Authors:** Iwan Harries, Giovanni Biglino, Kerrie Ford, Martin Nelson, Gui Rego, Prashant Srivastava, Matthew Williams, Bostjan Berlot, Estefania De Garate, Anna Baritussio, Kate Liang, Mai Baquedano, Nikesh Chavda, Christopher Lawton, Andrew Shearn, Sophie Otton, Lisa Lowry, Angus K. Nightingale, Juan Carlos Plana, David Marks, Costanza Emanueli, Chiara Bucciarelli-Ducci

**Affiliations:** a Bristol Heart Institute, Bristol Medical School, University Hospitals Bristol, Bristol, UK; b Myocardial Function – National Heart and Lung Institute, Imperial College London, London, UK; c NIHR Bristol Biomedical Research Centre, Bristol Heart Institute, University Hospitals Bristol NHS Foundation Trust, Bristol, UK; d Bristol Heamatology and Oncology Centre, University Hospitals Bristol NHS Trust, Bristol United Kingdom, UK; e North Bristol NHS Trust, Bristol, UK; f Musgrove Park Hospital, Taunton, UK; g Baylor College of Medicine, Houston, TX, United States; h Royal Brompton and Harefield Hospitals, Guys’ and St Thomas NHS Foundation Trust, London; i School of Biomedical Engineering and Imaging Sciences, Faculty of Life Sciences and Medicine, Kings College, London

**Keywords:** Cardio-oncology, Cancer therapeutics-related cardiac dysfunction, Cardiovascular magnetic resonance

## Abstract

**Background:**

Anthracycline cardiotoxicity is a significant clinical challenge. Biomarkers to improve risk stratification and identify early cardiac injury are required.

**Objectives:**

The purpose of this pilot study was to prospectively characterize anthracycline cardiotoxicity using cardiovascular magnetic resonance (CMR), echocardiography and MicroRNAs (MiRNAs), and identify baseline predictors of LVEF recovery.

**Methods:**

Twenty-four patients (age 56 range 18–75 years; 42 % female) with haematological malignancy scheduled to receive anthracycline chemotherapy (median dose 272 mg/m^2^ doxorubicin equivalent) were recruited and evaluated at three timepoints (baseline, completion of chemotherapy, and 6 months after completion of chemotherapy) with multiparametric 1.5 T CMR, echocardiography and circulating miRNAs sequencing.

**Results:**

Seventeen complete datasets were obtained. CMR left ventricular ejection fraction (LVEF) fell significantly between baseline and completion of chemotherapy (61 ± 3 vs 53 ± 3 %, p < 0.001), before recovering significantly at 6-month follow-up (55 ± 3 %, p = 0.018). Similar results were observed for 3D echocardiography-derived LVEF and CMR-derived longitudinal, circumferential and radial feature-tracking strain. Patients were divided into tertiles according to LVEF recovery (poor recovery, partial recovery, good recovery). CMR-derived mitral annular plane systolic excursion (MAPSE) was significantly different at baseline in patients exhibiting poor LVEF recovery (11.7 ± 1.5 mm) in comparison to partial recovery (13.7 ± 2.7 mm), and good recovery (15.7 ± 3.1 mm; p = 0.028). Furthermore, baseline miRNA-181-5p and miRNA-221-3p expression were significantly higher in this group. T2 mapping increased significantly on completion of chemotherapy compared to baseline (54.0 ± 4.6 to 57.8 ± 4.9 ms, p = 0.001), but was not predictive of LVEF recovery. No changes to LV mass, extracellular volume fraction, T_1_ mapping or late gadolinium enhancement were observed.

**Conclusions:**

Baseline CMR-derived MAPSE, circulating miRNA-181-5p, and miRNA-221-3p were associated with poor recovery of LVEF 6 months after completion of anthracycline chemotherapy, suggesting their potential predictive role in this context. T2 mapping increased significantly on completion of chemotherapy but was not predictive of LVEF recovery.

## Highlights

• LVEF partially recovers six months after anthracycline in the majority of patients.
• Baseline CMR derived MAPSE is associated with poor recovery of LVEF six months after completion of anthracycline.
• Baseline circulating levels of miRNA-181-5p and miRNA-221-3p associated with poor recovery of LVEF six months after completion of anthracycline.
• Multiple, significant temporal changes identified by CMR, echocardiography and biomarkers in response to anthracycline.

## 1 Introduction

Anthracycline provides effective treatment for several forms of cancer, but its use is mitigated by dose-dependent cardiotoxicity[1]. Consequently, societal guidelines recommend serial monitoring of left ventricular ejection fraction (LVEF) in patients receiving these regimens[2], [3], [4].

Mitochondrial edema and intracardiomyocyte vacuolization are the histopathological hallmarks of early anthracycline cardiotoxicity[5], [6] but importantly, these changes seem to predate myocardial functional changes[5]. Indeed, tissue level damage may already be extensive by the time a change in LVEF is detected. The later stages of cardiotoxicity are characterized by cell death, replacement fibrosis[7] and an adverse prognosis[8].

Contemporary dose-capped regimes mean that cardiotoxicity affects approximately 9 % of patients[9]. Furthermore, recovery of LVEF following anthracycline is unpredictable, with only a minority of patients returning to baseline systolic function[9]. Identifying cardiotoxicity early and instituting protective therapy reduces cardiovascular adverse events[10] and facilitates therapeutic efficacy. Notwithstanding the recognition of contributory clinical risk factors such as age, female sex, pre-existing cardiovascular disease[2], [4] and genetic predisposition[11], predicting individual susceptibility to anthracycline cardiotoxicity continues to pose a significant clinical challenge. All of which underscores the need for of an accurate, reproducible, non-invasive biomarker that can either identify cardiotoxicity at an early stage or identify patients whose LVEF may not recover.

Cardiovascular magnetic resonance (CMR) is the non-invasive reference standard method to assess cardiac structure and function[12], and the advent of multiparametric sequences furnish the unique ability to characterize myocardial tissue non-invasively. The ability of CMR to detect myocardial edema and fibrosis with native T_2_, and T_1_ mapping sequences, respectively was recently validated histopathologically in a porcine model of anthracycline cardiotoxicity[13]. However, to date, few adult human studies have used prospective CMR to catalogue the cardiac effects of anthracycline[14], [15], [16], [17], [18], [19] and fewer still have included multiparametric mapping techniques[20], [21], [22], [23], [24].

Circulating miRNAs are short non-coding RNAs that regulate cellular processes and play a critical role in cardiovascular biology[25]. Their relative stability and ease of quantification, combined with high levels of sensitivity and specificity, make them an appealing biomarker in several cardiovascular disease states, with emerging animal[26] and latterly human models[27], [28] reporting temporal dysregulation in the context of anthracycline cardiotoxicity.

Accordingly, the objective of this pilot study was to prospectively characterize the cardiac effects of anthracycline using multiparametric CMR, echocardiography and miRNAs and to explore predictors of LVEF recovery following completion of treatment.

## 2 Methods

### 2.1 Study design and participants

From April 2017 to August 2018, we prospectively enrolled 24 patients at two haematology centres in Bristol, United Kingdom: Bristol Haematology and Oncology Centre and Southmead Hospital. The study was approved by the West Midlands - Solihull Research Ethics Committee. All participants provided written informed consent.

The study enrolled consecutive patients age > 18 with a new diagnosis of high-grade non-Hodgkin (NHL) and Hodgkin lymphoma (HL) scheduled for ≥ 6 cycles of anthracycline, or acute myeloid leukaemia (AML) scheduled for full dose treatment with curative intent. Patients were not eligible if they had a history of prior cardiac disease or malignancy, prior chemotherapy, or had contraindications to CMR (ferromagnetic foreign body, claustrophobia, severe renal impairment [eGFR < 30 ml/min/1.73 m^2^]).

**Fig. 1:**
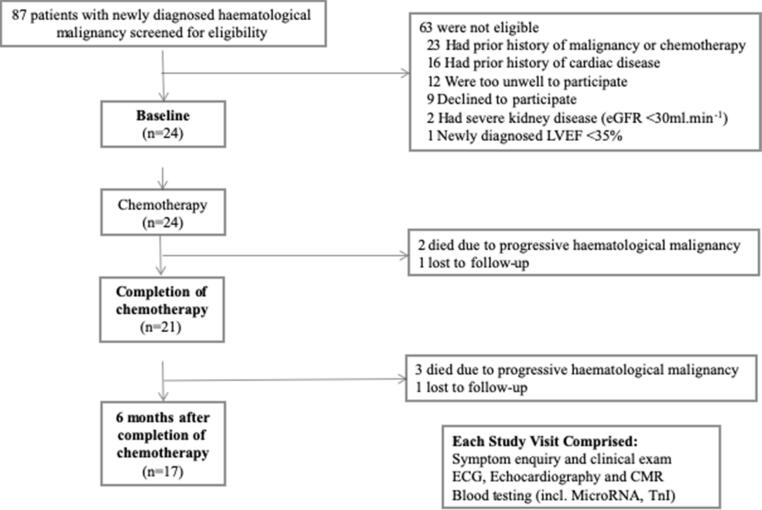
Study enrolment and follow-up.

CMR, transthoracic echocardiography, electrocardiogram (ECG), serum biomarkers (including miRNAs and Troponin I [TnI]) and symptom enquiry were performed on the same day at three time points: prior to chemotherapy (baseline), on completion of chemotherapy, and 6 months after completion of chemotherapy. Three patients were withdrawn between baseline and completion of chemotherapy (2 deaths, 1 lost to follow-up) and another four between completion of chemotherapy and 6-month follow-up (3 deaths, 1 lost to follow-up; Fig. 1), giving a total of 17 patients with complete assessments. In secondary analysis, these 17 patients were divided into tertiles according to recovery of LVEF between completion of chemotherapy and 6-month follow-up i.e. the 6 patients exhibiting least LVEF recovery were placed in the bottom tertile. Predictors of LVEF recovery were explored from baseline and completion of chemotherapy visits.

The research team designed the study, gathered and analysed the data, prepared the manuscript for publication and vouch for data fidelity. Blinded, random order analysis of anonymised imaging data was undertaken at the end of the study. Data was collected and managed using Research Electronic Data Capture (REDCap)[29], hosted at Bristol University Hospitals NHS Foundation Trust.

### 2.2 Cardiovascular magnetic resonance

#### 2.2.1 Left ventricular volumes, ejection fraction and strain

Patients underwent CMR at 1.5 Tesla (Avanto, Siemens, Erlangen, Germany). Short-axis steady state free precession cines (typical parameters: 8 mm slice thickness [no gap], temporal resolution 38.1 ms, echo time 1.07 ms, in-plane pixel size 1.5 × 0.8 mm) of the left ventricle were used to measure left ventricular (LV) volumes, ejection fraction (EF) and mass by drawing epicardial and endocardial borders at end-systole and end-diastole. Measurements were indexed to body surface area, according to established methods[30]. Average global longitudinal, circumferential and radial strain parameters were calculated from short and long (two, three and four chamber) axis cines using voxel-tracking post-processing software (CVI 42, Circle Cardiovascular Imaging, Calgary, Canada). Lateral mitral annular plane systolic excursion (MAPSE) was measured manually in the four-chamber view.

#### 2.2.2 Native myocardial T_1_ mapping, T_2_ mapping and ECV

A modified look-locker inversion (MOLLI) recovery sequence[31] (35° flip angle, 100 ms minimum TI, 80 ms TI increment, 150 ms time delay with 5-(3)-3 heartbeat acquisition scheme) was used to obtain a motion-corrected, short axis native myocardial T_1_ map of the mid ventricular septum, on which a region of interest was drawn to determine native myocardial T_1_ measurements. A blood sample was taken immediately prior to CMR to determine TnI and haematocrit (Abbott Laboratories, United States). The latter allowed calculation of ECV using the established formula[32]: ECV = (1-haematocrit) × (ΔR1 _myocardium_/ΔR1_blood_). Short axis mid ventricular T_2_ maps were obtained using a T_2_ prepared steady state free precession sequence (223.77/1.12; 70° flip angle, section thickness 8 mm; field of view, 340–400 mm; matrix 156x192; voxel size 2.3 × 1.9 × 8 mm), on which a region of interest was drawn to determine native myocardial T_2_ measurements, using Argus software (Siemens Healthineers, Germany).

### 2.3 Echocardiography

#### 2.3.1 Left ventricular volumes, ejection fraction (2D and 3D) and global longitudinal strain

Echocardiograms were performed by two fully-accredited researchers using a single Epiq machine (Philips, Netherlands). Biplane 2D LVEF was calculated using Simpson’s rule from apical four and two chamber images and 3D LVEF using semi-automated software (HeartModel, Philips, Netherlands). Global longitudinal strain (GLS) was measured by tracing three fiducial landmarks (apex, lateral and medial mitral annulus) on the endocardial border with automated function imaging (Automated Cardiac Motion Quantification [aCMQ], Philips, Netherlands). The fidelity of contours to cardiac motion was tracked through the cardiac cycle and manually adjusted, if necessary. Measures were not recorded if there was inadequate visualization of ≥ 3 contiguous myocardial segments. MAPSE was measured in the four chamber view at the lateral mitral annulus.

### 2.4 Electrocardiography

A 12 lead surface electrocardiogram (ECG) was obtained at each study visit and assessed for abnormalities of rhythm, conduction intervals, ST and *T*-wave morphology.

### 2.5 Serum Troponin I and miRNAs

Blood was drawn at each study visit. Troponin I (TnI) was determined at the bedside (Abbott Laboratories, United States). Platelet-poor plasma was prepared by drawing blood into a sodium citrate vacutainer and centrifuging at 2240 g for 10 min at room temperature. The plasma supernatant was removed to a fresh tube without disturbing the buffy coat and centrifuged again at 2240 g for 10 min at room temperature. The supernatant was removed to a fresh Eppendorf LoBind microcentrifuge tube and stored at −80 °C[33]. RNA extraction, small RNA sequencing and data quality control and alignment were performed on completion of the study by Qiagen (Germany) on samples obtained prior to, and on completion of chemotherapy. Differential expression analysis (likelihood ratio test following false discovery rate (FDR) correction) comparing circulating miRNA expression in patients in tertile 1 (poor LVEF recovery) with tertile 3 (good LVEF recovery) was performed in R using the edgeR package[34], [35]. A miRNA was considered to be significantly altered if FDR was less than0.05.

### 2.6 Data analysis

Data from the 17 patients who completed all visits were divided into tertiles according to LVEF recovery between completion of chemotherapy and 6-month follow-up. Predictors of LVEF recovery were explored from baseline and completion of chemotherapy visits. Overall data are presented as mean ± standard deviation for continuous variables; counts and percentages for categorical variables. Paired analysis (Wilcoxon signed rank test) was used to assess temporal changes. Following rejection of a Kruskal-Wallis test, post hoc Dunn’s pairwise comparison was used in secondary analysis to explore predictors of left ventricular recovery, comparing tertiles, as defined above. Associations between variables were assessed with linear regression. Based on the available literature[14], [16], [17], we calculated that a significant drop in LVEF of 6.2 ± 3 % could be captured with a sample of 14 patient compared over 3 timepoints (alpha = 0.016, power = 0.9). Other elements of the work (e.g.MiRNA) remain exploratory. Ultimately, a sample of 25 patients was targeted accounting for 20 % dropout (10 % mortality, 10 % loss to follow-up). Statistical analysis was performed using Stata (Stat version 13, StatCorp LLC, United States) with a p value of < 0.05 considered significant.

## 3 Results

### 3.1 Patient characteristics

Baseline demographic data of all participants are displayed in Table 1. The mean age was 56 years (range 18 to 75). One patient was newly diagnosed with one segment of subendocardial late gadolinium enhancement (LGE) in keeping with previously unrecognized myocardial infarction at baseline. All patients received anthracycline at a mean total doxorubicin equivalent dose of 272 mg/m^2^ (range 112 to 412). Nineteen patients (79 %) received a monoclonal antibody, and fourteen (58 %) received either prednisolone or methylprednisolone during the course of their treatment.

**Table 1:** Baseline data.

	(N = 24)
Mean age (range) - years	56 (18–75)
Sex – no. (%) Male Female	14 (68)10 (42)
Race White Mixed-race (Black/White) Middle Eastern	22 (92)1 (4)1 (4)
Mean body surface area (m^2^)	1.91 ± 0.22
Mean body mass index (kg/m^2^)	26.1 ± 4.8
NYHA Class I – no. (%)	24 (1 0 0)
Medical history – no. (%) Hypertension Diabetes Dyslipidaemia Current smoker Ex-smoker Mean alcohol intake (range) - units/week	5 (21)2 (8)5 (21)6 (25) 8 (33)4 (0–20)
Cardiovascular medications – no. (%) Angiotensin converting enzyme inhibitor Angiotensin II receptor blocker Beta-blocker Statin	1 (4)1 (4)1 (4)5 (21)
Haematological diagnosis – no. (%) Acute myeloid leukaemia Non-Hodgkin lymphoma Hodgkin lymphoma	9 (38)12 (50)3 (13)
Chemotherapy – no. (%) Anthracycline Idarubicin Daunorubicin Doxorubicin Mean Doxorubicin equivalent dose (range) - mg/m^2^ Monoclonal antibody Rituximab Gemtuzumab Alemtuzumab Cyclophosphamide Prednisolone/methylprednisolone	24 (1 0 0)3 (13)6 (25)15 (63) 272 (112–412)19 (79)12 (50)5 (21)4 (17)12 (50)14 (58)
Radiotherapy – no. (%) Cumulative radiation dose (Gy)	2 21 ± 13

Plus-minus values are means ± SD.

### 3.2 Cardiovascular magnetic resonance

Indexed left ventricular end-diastolic volumes (iLVEDV) did not change between any timepoint (Table 2). Indexed left ventricular end systolic volumes increased significantly between baseline (31 ± 11 ml/m^2^) and completion of chemotherapy (35 ± 9 ml/m^2^, p < 0.001), and decreased significantly between completion of chemotherapy and 6-month follow-up (32 ± 6 ml/m^2^, p = 0.014), with no difference between baseline and 6-month follow-up (p = 0.89). LVEF was normal (61 ± 3 %) at baseline, decreased significantly on completion of chemotherapy (53 ± 3 %, p < 0.001), increased significantly at 6-month follow-up (55 ± 3 %, p = 0.018) but remained significantly decreased compared to baseline (p < 0.001). Mitral annular plane systolic excursion (MAPSE) decreased significantly from baseline to 6-month follow-up (14.0 ± 2.6 to 12.5 ± 2.4 mm, p = 0.048) and RVEF also decreased significantly between baseline (60 ± 6 %) to completion of chemotherapy (56 ± 6, p = 0.005). One patient met criteria for cardiotoxicity (>10 % absolute percentage drop in LVEF to a value < 53 %[2]) on completion of chemotherapy, which recovered by 6-month follow-up.

**Table 2:** Temporal changes in CMR, echocardiographic, serum and physiological metrics.

	Visit 1 N = 24	Δ V1-V2 p value	Visit 2 N = 21	Δ V2-V3 p value	Visit 3 N = 17	Δ V1-3p value
CMR iLVEDV (ml/m2) iLVESV (ml/m2) LVEF (%) MAPSE (mm) LAVi (ml/m2) LVMi RVEF (%) 2D FTGLS (%) 2D FTGCS (%) 2D FTGRS (%) Myocardial T_1_ (ms) Myocardial T_2_ (ms) ECV (%)	77 ± 19 31 ± 11 61 ± 3 14.0 ± 2.6 45 ± 12 46 ± 9 60 ± 6 −21.1 ± 3.1 −23.7 ± 3.3 55.2 ± 11.6 1048 ± 33 54.0 ± 4.6 29.4 ± 3.4	0.24 **0.02** **<0.001** 0.17 0.10 0.52 **0.005** **<0.001** **<0.001** **<0.001** 0.348 **0.001** 0.40	74 ± 15 35 ± 9 53 ± 3 13.0 ± 3.0 41 ± 10 47 ± 7 56 ± 6 −17.8 ± 2.5 −21.1 ± 3.5 44.7 ± 8.8 1055 ± 35 57.8 ± 4.9 30.3 ± 3.9	0.30 **0.014** **0.018** 0.89 0.11 0.16 0.37 **0.02** **0.02** **0.03** 0.91 0.14 0.11	72 ± 14 32 ± 6 55 ± 3 12.5 ± 2.4 43 ± 9 44 ± 7 59 ± 6 −19.0 ± 2.6 −22.5 ± 2.8 49.9 ± 9.0 1055 ± 36 55.3 ± 3.7 28.2 ± 4.7	0.17 0.89 **<0.001** **0.048** 0.47 0.22 0.28 **0.002** 0.255 0.09 0.61 0.13 0.39
Echocardiography 2D LVEF (%) 3D LVEF (%) GLS (%)	62 ± 3 61 ± 4 −20.6 ± 2.6	0.18 **0.001** 0.12	60 ± 7 57 ± 4 −19.2 ± 3.6	0.939 **0.035** 0.56	59 ± 4 60 ± 4 −18.9 ± 2.1	0.11 0.81 0.053
Serum TnI (ng/ml) Hb (g/dL) Haematocrit (%) eGFR (ml/min/1.73 m^2^) CRP	0.004 ± 0.013 11.8 ± 2.7 35 ± 8 83 ± 27 35 ± 57	**0.02** 0.112 **0.02** 0.12 0.11	0.023 ± 0.030 11.0 ± 2.5 26 ± 15 94 ± 31 12 ± 14	0.06 **0.02** **0.046** 0.321 0.70	0.07 ± 0.06 12.9 ± 2.2 36 ± 11 104 ± 55 17 ± 31	0.57 0.10 0.93 0.65 **0.03**
Physiology Body mass (kg) HR (bpm) SBP (mmHg) DBP (mmHg) NYHA I (%) NYHA II NYHA III NYHA IV	76 ± 15 79 ± 12 131 ± 15 78 ± 9 22 (91)2 (9)0 (0)0 (0)	0.72 0.55 0.29 0.40	74 ± 14 74 ± 9 128 ± 12 75 ± 12 14 (66)7 (33)0 (0)0 (0)	0.55 0.95 0.12 0.19	75 ± 16 74 ± 8 123 ± 22 72 ± 13 11 (65)5 (29)1 (6)0 (0)	0.80 0.23 0.16 **0.019**

Values are mean ± standard deviation or n (%). 2D = two dimensional, CMR = cardiovascular magnetic resonance, CRP = C-reactive protein, DBP = diastolic blood pressure, ECV = extracellular volume fraction, FT = feature tracking, GLS = global longitudinal strain, GCS = global circumferential strain, GRS = global radial strain, Hb = haemoglobin, HR = heart rate, LAVi = left atrial volume indexed, LVEDVi = left ventricular end diastolic volume indexed, LVESVi = left ventricular end systolic volume indexed, LVEF = left ventricular ejection fraction, LVMi = left ventricular mass indexed, MAPSE = mitral annular plane systolic excursion, RVEF = right ventricular ejection fraction, SBP = systolic blood pressure, T_1_ = T_1_ relaxation time, T_2_ = T_2_ relaxation time, TnI = troponin I, eGFR = estimated glomerular filtration rate.

All measures of 2D feature tracking (FT) CMR strain worsened significantly on completion of chemotherapy, compared to baseline (p < 0.001 in all cases; Table 2). All measures of 2D FT strain worsened significantly between completion of chemotherapy and 6-month follow-up (p < 0.05), but of all FT CMR strain parameters, only 2D GLS was persistently impaired at 6-month follow-up (-19.0 ± 2.6 %), compared to baseline (-21.1 ± 3.1, p = 0.002).

No significant temporal changes in native myocardial T_1_ measurements or ECV were observed during the study (Table 2). Native myocardial T_2_ measurements increased significantly from baseline to completion of chemotherapy (54.0 ± 4.6 to 57.8 ± 4.9 ms, p = 0.001) but no other significant between-visit differences were recorded. Furthermore, neither the absolute native myocardial T_2_, nor the change in native myocardial T_2_ between baseline and completion of chemotherapy correlated with any parameters of left ventricular systolic function. No new LGE was observed during the study. Indexed left ventricular mass did not change significantly ***(***Table 2***).***

### 3.3 Echocardiography

Echo-derived 2D LVEF was not significantly different across study visits. However, 3D LVEF decreased significantly between baseline and completion of chemotherapy (61 ± 4 to 57 ± 4 %, p = 0.001) and increased significantly between completion of chemotherapy and 6-month follow-up (57 ± 4 to 60 ± 4 %, p = 0.035), though there was no significant difference between baseline and 6-month follow-up (p = 0.81). Echo-derived GLS did not differ significantly across study visits, though there was a trend towards worsened GLS from baseline to 6-month follow-up (-20.6 ± 2.6 to −18.9 ± 2.1 %, p = 0.053).

### 3.4 Electrocardiography

One patient had ECG evidence of old anteroseptal myocardial infarction at baseline. No changes to rhythm, conduction intervals or ST segments or *T*-wave morphology were detected during the study.

### 3.5 Troponin

Troponin I increased significantly between baseline and completion of chemotherapy (4.3 ± 13.2 to 22.9 ± 29.9 pg/ml, p = 0.02) but did not otherwise differ. Three patients (14 %) exhibited subclinical cardiac injury (TnI of 60.0 to 100.0 pg/ml) on completion of chemotherapy.

### 3.6 Cardiovascular physiology and symptoms

The only significant change to physiological metrics during the course of the study was that diastolic blood pressure decreased significantly between baseline and 6-month follow-up (78 ± 9 to 72 ± 13 mmHg, p = 0.019; Table 2***)***. At baseline, 91 % (22/24) of patients were NYHA class I. This had fallen to 66 % (14/21) by completion of chemotherapy and 65 % (11/17) by 6-month follow-up. Seven patients (33 %) and five patients (33 %) reported NYHA class II dyspnea at completion of chemotherapy and 6-month follow-up, respectively. One patient (6 %) was NYHA class III at 6-month follow-up.

### 3.7 Recovery of left ventricular ejection fraction

**Fig. 2:**
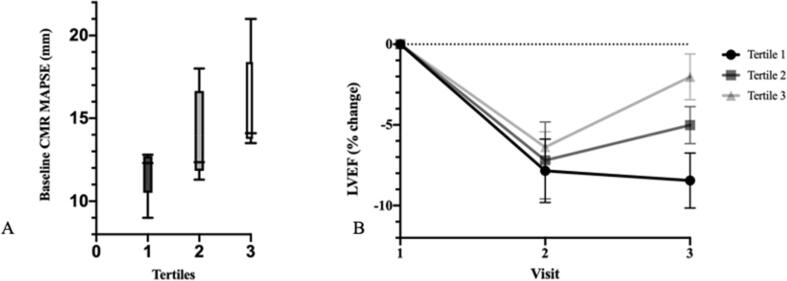
Panel A: Baseline CMR MAPSE according to tertile of LVEF recovery; Panel B temporal absolute % change in LVEF by tertile.

Patients were divided into tertiles according to CMR LVEF recovery between completion of chemotherapy and 6-month follow-up ***(***Fig. 2***).*** Selected baseline demographic and study data by tertile are displayed in Table 3. Patients in tertile 1 (poor recovery) trended towards being older than those in tertile 2 (partial recovery) and tertile 3 (good recovery), though this difference was not significant (p = 0.11). Baseline CMR-derived MAPSE was significantly different among tertiles of LVEF recovery, measuring 11.7 ± 1.5 mm, 13.7 ± 2.7 mm, 15.7 ± 3.1 mm in tertiles 1, 2 and 3, respectively (Fig. 2***;*** p = 0.028). Baseline myocardial T_1_ was also noted to be significantly different between tertiles, but measures were not ordinal, with tertile 2 exhibiting the highest values at baseline (Table 3***)***. No other baseline study data, nor change () between baseline and completion of chemotherapy study visits were significantly different between tertiles of LVEF recovery. Echocardiography and CMR-derived MAPSE correlated significantly (p < 0.001) but baseline echocardiography-derived MAPSE was not significantly associated with LVEF recovery (p = 0.32).

**Table 3:** Baseline data by LVEF recovery tertiles.

Values are mean (median) ± standard deviation or n (%). 2D = two dimensional, 3D = three dimensional, BSA = body surface area, CMR = cardiovascular magnetic resonance, Dox = Mean doxorubicin equivalent dose, FT = feature tracking, GLS = global longitudinal strain, GCS = global circumferential strain, GRS = global radial strain, LVEF = left ventricular ejection fraction, MAPSE = mitral annular plane systolic excursion, T_1_ = T_1_ relaxation time, T_2_ = T_2_ relaxation time, TnI = troponin I.

**Fig. 3:**
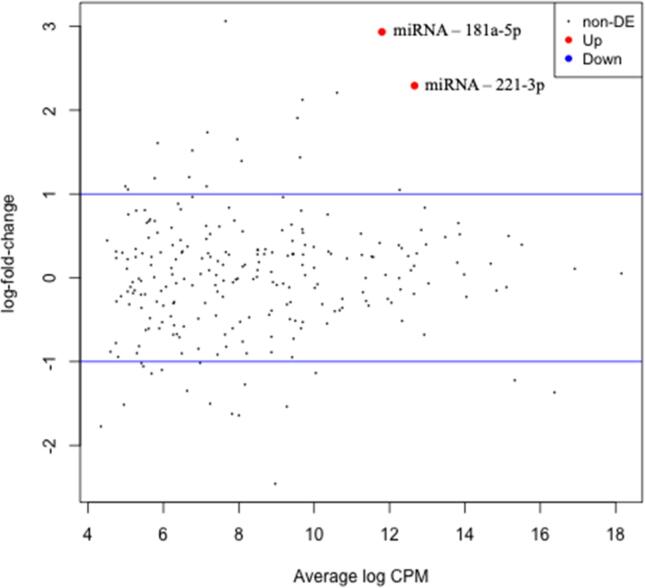
Dysregulation of miRNAs at baseline according to tertile of LVEF recovery. X axis represents the count per million of each miRNA in the sequencing, reflecting relative expression levels in plasma. Y axis represents the difference in expression between tertile 1 (poor recovery) and tertile 3 (good recovery). miRNA 181a-5p and miRNA 221-3p were upregulated at baseline in patients with poor recovery of lV ∼ EF, relative to those with good LVEF recovery. CPM = count per million, DE = dysregulated expression.

In analysis of baseline miRNAs according to tertiles of LVEF recovery, two miRNAs were significantly dysregulated (likelihood ratio test) after FDR correction: miRNA-181-5p and miRNA-221-3p (Fig. 3***)***. Both were expressed to a significantly higher degree in tertile 1, whose LVEF did not recover between completion of chemotherapy and 6-month follow-up. No significant dysregulation of miRNAs was detected on completion of chemotherapy according to patient groups defined by tertiles of LVEF recovery.

### 3.8 Discussion

In this prospective, multiparametric CMR, echocardiography and blood biomarker study of anthracycline cardiotoxicity, we report five important findings. First, multiple measures of left ventricular systolic function (CMR LVEF; CMR FT GLS, GCS and GRS; echo 3D LVEF) deteriorate significantly on completion of anthracycline treatment compared to baseline. Second, several measures of systolic function recover over the next 6 months, with only CMR LVEF, CMR MAPSE, and CMR 2D FTGLS persistently depressed. Third, native myocardial T_2_ mapping elevation occurred following anthracycline treatment but was not predictive of subsequent LVEF recovery. Fourth, no changes in native myocardial T_1_, ECV, LGE or LV mass were recorded in our study with the caveat that only one patient met criteria for cardiotoxicity during the course of the study. Fifth, analysis of LVEF recovery by tertile revealed that CMR MAPSE was significantly different at baseline in patients exhibiting poor recovery of LVEF following completion of anthracycline. Furthermore, baseline expression of miRNA-181-5p and miRNA-221-3p was significantly higher in those with poor LVEF recovery.

#### 3.8.1 Cardiovascular magnetic resonance volumes and systolic function

Commensurate with previous prospective studies of anthracycline cardiomyopathy[14], [20], we observed significant reductions in LVEF early after completion of anthracycline treatment, which were driven by an increase in iLVESV (iLVEDV did not change). The reduction in LVEF following anthracycline treatment was relatively uniform across the entire cohort but recovery of LVEF over the following 6 months exhibited significant variation, Indeed, we observed only partial recovery of LVEF 6 months after anthracycline treatment and built on previous reports describing similar trends[22], [23] by exploring baseline and early predictors of LVEF recovery by dividing the populations into tertiles of LVEF recovery. CMR-derived MAPSE was the only baseline imaging metric significantly associated with LVEF recovery; which is a novel finding. Long-axis systolic motion of the mitral annulus is an integral component of cardiac mechanics and the utility of CMR-derived MAPSE as a powerful independent predictor of cardiovascular outcomes has recently been reported in the context of hypertension[36] and myocardial infarction[37]. Indeed, it is reported that long axis motion (for which MAPSE is a surrogate) is responsible for approximately 60 % of stroke volume[38]. Furthermore, MAPSE can be calculated quickly and easily from standard cine images, without the need for specialized sequences. Therefore, the role of CMR-derived MAPSE as a baseline predictor of LVEF recovery after anthracycline treatment may potentially be of clinical interest in selecting patients for more frequent monitoring or the institution of preventive therapies, though confirmation by larger, adequately powered prospective studies is required. It is also noteworthy that despite the administration of comparatively high doses of anthracycline to a patient cohort with coexisting haematological malignancy and increasing breathlessness (judged by NYHA class), only one patient met criteria for cardiotoxicity during the course of the study, highlighting the fact that in the vast majority of patients, the observed changes were subclinical.

#### 3.8.2 Cardiovascular magnetic resonance strain

Our study prospectively documented anthracycline-related changes in 2D FT-strain in all three planes and as such, provides novel insight. All measures of CMR strain were significantly impaired on completion of anthracycline treatment. However, FTGCS and FTGRS were statistically comparable to baseline by the end of the study, whereas FTGLS remained persistently impaired. These data support and replicate previous work by Ong et al.[19] who reported similar finding for FTGLS and FTGCS in a prospectively studied cohort of breast cancer patients treated with trastuzumab, half of whom also received anthracycline. Measures of strain at baseline and on completion of chemotherapy were not associated with subsequent LVEF recovery. It is unclear why longitudinal strain wither by CMR or echocardiography did not predict recovery of LVEF but possible explanations include the reproducibility of strain measures, as well as the small sample size.

#### 3.8.3 Cardiovascular magnetic resonance T_1_ and T_2_ mapping

Recent animal data demonstrated that T_2_ mapping abnormalities are the earliest marker of cardiotoxicity, which manifested histopathologically as intracardiomyocyte vacuolisation[13]. Importantly, cessation of anthracycline at this stage led to resolution of imaging and histological changes, whereas continuation led to progressive abnormalities of imaging, histological and functional markers, thus potentially identifying cardiotoxicity at a reversible stage[13]. In turn, human studies have reported significant [39], [40] and non-significant[20] elevation of T_2_ mapping early after anthracycline chemotherapy, though their relationship to subsequent cardiac dysfunction is hampered by short duration of follow up[20], [39] and lack of sequential data collection[40]. Our study provides additional evidence of significant elevation of T_2_ mapping early after chemotherapy, with prospective evaluation confirming resolution of T_2_ mapping over a longer period of follow-up. However, early T_2_ mapping elevation was not associated with subsequent LVEF recovery, either on a grouped or individual level in our study.

The potential role of native T_1_ mapping and ECV in the detection of early cardiotoxicity have been highlighted by animal[26] and human[20], [22], [39], [40] studies describing significant temporal changes. In contrast, we found no significant changes to T_1_ mapping or ECV during the course of the study. This discrepancy may partly relate to the differing imaging timing and populations studied (ours is the only study exclusively of haematological malignancies), the low incidence of cardiotoxicity, as well as the confounding effects of potent anti-inflammatory treatment (58 % received concomitant steroid in our study; not reported by others) and cancer itself[41] on native T_1_ mapping and ECV. When taken in combination with a recent report of overlapping temporal variability between patients and healthy controls[42], the precise translational role for these sequences in modern monitoring strategies is yet to be defined. The reduced sensitivity native T1 mapping in comparison to T2 may explain why n temporal change in native T1 mapping values was observed.

#### 3.8.4 Echocardiography

Echocardiography is the most accessible and widespread method to monitor cardiac function during anthracycline therapy[3]. Despite changes in multiple CMR metrics, only 3D LVEF changed significantly in our study, in keeping with a previous report highlighting the relative lack of sensitivity of 2D methods to detect temporal changes in LVEF[43]. Furthermore, no baseline or early echocardiographic predictors of LVEF recovery were identified in our study. The absence of predictive value in echo derived MAPSE may relate to dependence on imaging angle and quality with the former, which do not typically affect CMR-derived MAPSE.

#### 3.8.5 MicroRNAs

Circulating miRNAs regulate target gene expression and are an appealing biomarker because they are readily quantifiable, have a long half-life, and remain stable at extremes of temperature and pH. However, few human studies have examined their utility to detect anthracycline cardiotoxicity, and have reported a range of expression profiles[27], [28], [44]. In our study analysing predictors of LVEF recovery after completion of anthracycline, we identified that miRNA-181a-5p and miRNA-221-3p were significantly increased at baseline in patients whose LVEF showed poor recovery after anthracycline chemotherapy. A recent systematic review and *meta*-analysis reported that miRNA-221 was associated with a poor overall survival in human carcinoma[45] and miRNA 181a-5p expression has been associated with poor outcomes in patients with colorectal cancer[46] but their role in anthracycline cardiotoxicity has not previously been reported. Therefore, it is uncertain whether increased MiRNAs levels at baseline signify susceptibility to cardiotoxicity, or are a broader marker of malignancy with an adverse prognosis. Indeed subclinical functional and morphological cardiac dysfunction has previously been linked to cancer itself[47], [48], independent of chemotherapy and further work is required to explore these relationships.

From a methodological stand point, this study can also represent a model for designing future translation studies exploring both imaging and circulation blood biomarkers in this patients’ population.

### 3.9 Study limitations

This study intentionally recruited a homogenous cohort of patients to minimize baseline confounding factors and used specific hardware and software to collect and analyse data, which limits the external validity of the results to other populations and clinical systems. This was a pilot study, powered to detect temporal changes in LVEF and as such, secondary analysis for baseline and early predictors of LVEF recovery are to be considered exploratory and require further validation before firm conclusions can be drawn. Furthermore, small sample sizes are subject to both type I and type II errors, underscoring the need for validation by larger studies before firm conclusions can be drawn.The low incidence of cardiotoxicity and absence of major adverse cardiovascular events over a relatively short period of follow-up mean that the impact of the observed subclinical temporal changes on clinical outcomes has not been established.

## 4 Conclusions

In this prospective multimodality pilot study, we demonstrated several temporal multiparametric CMR, echocardiographic and serological cardiac changes in response to anthracycline chemotherapy with varying recovery of LVEF in the 6 months following completion of treatment. Baseline CMR-derived MAPSE and baseline circulating miRNA-181-5p, and −221-3p were associated with poor recovery of LVEF 6 months after completion of anthracycline chemotherapy. Pending validation by larger studies, these potential ‘predictive’ biomarkers may be a useful tool to inform individualized treatment and monitoring strategies for patients scheduled to receive anthracycline treatment.

## Declaration of Competing Interest

The authors declare that they have no known competing financial interests or personal relationships that could have appeared to influence the work reported in this paper.
