# Supplementary material for: Prospective multiparametric CMR characterization and MicroRNA profiling of anthracycline cardiotoxicity: A pilot translational study
Source: Int J Cardiol Heart Vasc. 2022 Nov 8;43:101134. doi: 10.1016/j.ijcha.2022.101134 (PMC9647504; doi:10.1016/j.ijcha.2022.101134)
Supplement: Supplementary data 1 [file mmc1.docx]

**Table 1. Baseline data**

|  | (N =24) |
| --- | --- |
| Mean age (range) - years | 56 (18-75) |
| Sex – no. (%)  Male  Female | 14 (68)  10 (42) |
| Race  White  Mixed-race (Black/White)  Middle Eastern | 22 (92)  1 (4)  1 (4) |
| Mean body surface area (m^2^) | 1.91 ± 0.22 |
| Mean body mass index (kg/m^2^) | 26.1 ± 4.8 |
| NYHA Class I – no. (%) | 24 (100) |
| Medical history – no. (%)  Hypertension  Diabetes  Dyslipidaemia  Current smoker  Ex-smoker  Mean alcohol intake (range) - units/week | 5 (21)  2 (8)  5 (21)  6 (25)  8 (33)  4 (0-20) |
| Cardiovascular medications – no. (%)  Angiotensin converting enzyme inhibitor  Angiotensin II receptor blocker  Beta-blocker  Statin | 1 (4)  1 (4)  1 (4)  5 (21) |
| Haematological diagnosis – no. (%)  Acute myeloid leukaemia  Non-Hodgkin lymphoma  Hodgkin lymphoma | 9 (38)  12 (50)  3 (13) |
| Chemotherapy – no. (%)  Anthracycline  Idarubicin  Daunorubicin  Doxorubicin  Mean Doxorubicin equivalent dose (range) - mg/m^2^  Monoclonal antibody  Rituximab  Gemtuzumab  Alemtuzumab  Cyclophosphamide  Prednisolone/methylprednisolone | 24 (100)  3 (13)  6 (25)  15 (63)  272 (112 - 412)  19 (79)  12 (50)  5 (21)  4 (17)  12 (50)  14 (58) |
| Radiotherapy – no. (%)  Cumulative radiation dose (Gy) | 2  21 ± 13 |
| Plus-minus values are means ± SD. | |
